# An efficient gene disruption method for the woody plant pathogen *Botryosphaeria dothidea*

**DOI:** 10.1186/s12896-020-00608-z

**Published:** 2020-03-05

**Authors:** Bao-Zhu Dong, Li-Yun Guo

**Affiliations:** 1 Laboratory of Mycology, College of Plant Protection, China Agricultural University, Beijing, 100193 China; 2 Key Laboratory of Pest Monitoring and Green Management, College of Plant Protection, China Agricultural University, Beijing, 100193 China

**Keywords:** *Botryosphaeria dothidea*, Tree pathogen, Gene disruption, Homologous recombination

## Abstract

**Background:**

*Botryosphaeria dothidea* causes apple white rot and infects many tree plants. Genome data for *B. dothidea* are available and many pathogenesis-related genes have been predicted. However, a gene manipulation method is needed to study the pathogenic mechanism of *B. dothidea*.

**Results:**

We established a gene disruption (GD) method based on gene homologous recombination (GHR) for *B. dothidea* using polyethylene glycol-mediated protoplast transformation. The results showed that a GHR cassette gave much higher GD efficiency than a GHR plasmid. A high GD efficiency (1.3 ± 0.14 per 10^6^ protopasts) and low frequency of random insertions were achieved with a DNA cassette quantity of 15 μg per 10^6^ protoplasts. Moreover, we successfully disrupted genes in two strains. *Bdo_05381*-disrupted transformants produced less melanin, whereas the *Bdo_02540*-disrupted transformant showed a slower growth rate and a stronger resistance to Congo red.

**Conclusion:**

The established GD method is efficient and convenient and has potential for studying gene functions and the pathogenic mechanisms of *B. dothidea* and other coenocytic fungi.

## Background

*Botryosphaeria dothidea* is a pathogen found worldwide that can infect hundreds of woody plant species [1], including apple, peach [2], eucalyptus [3], grape [4], pecan [5], and blueberry [6], and cause fruit rot, leaf spot, twig dieback, stem and branch canker, and tree death [7]. Apple white rot caused by *B. dothidea* is one of the most destructive diseases in China [8]. *B. dothidea* is a coencytic fungus. Colony in culture is whitish at beginning, and changes to olivaceous, dark grey, and black in reverse as it ages. In nature, it reproduces commonly through producing conidia contained in pycnidia, and occasionally through producing ascospores [1]. Pycnidia produced on culture are also dark colored due to the melanin accumulation. Melanin can protect organisms from environmental stress [9].

*B. dothidea* has a genome size of 43–45 Mb [1, 10–12]. Although the genomic sequence of *B. dothidea* is available and many pathogenesis-related genes have been predicted, its pathogenic mechanism is still unclear. This is mainly due to the lack of an efficient genetic manipulation method for this pathogen.

Commonly used methods for investigating gene functions include gene disruption (GD) through homologous recombination (gene targeting) [13], gene mutagenesis through T-DNA insertion [14–16], gene editing through CRISPR/Cas9 [17] and gene silencing through RNAi [18]. Among these methods, GD through homologous recombination is the most commonly used for studying gene functions in filamentous phytopathogenic fungi, including *Fusarium graminearum* [19], *Verticillium dahliae* [20], and *Magnaporthe oryzae* [21]. The power of GD by homologous recombination is that researchers can choose precisely both the gene to disrupt and the specific change to introduce [22]. Recently, polyethylene glycol (PEG)-mediated protoplast transformation and *Agrobacterium tumefaciens*-mediated transformation methods have been established for *B. dothidea* [23, 24]*,* but an efficient GD method remains to be developed.

In this study, we selected two genes, *Bdo_05381* (a predicted pheromone precursor protein) and *Bdo_02540* (a hypothetical protein), which were up-regulated during the process of infection and the melanin accumulation [25], as targets to develop the disruption method. We successfully established a convenient and efficient protocol for GD based on gene homologous recombination (GHR) for *B. dothidea* through PEG-mediated protoplast transformation.

## Results

### Using a GHR cassette for transformation gives higher GD efficiency

**Fig. 1:**
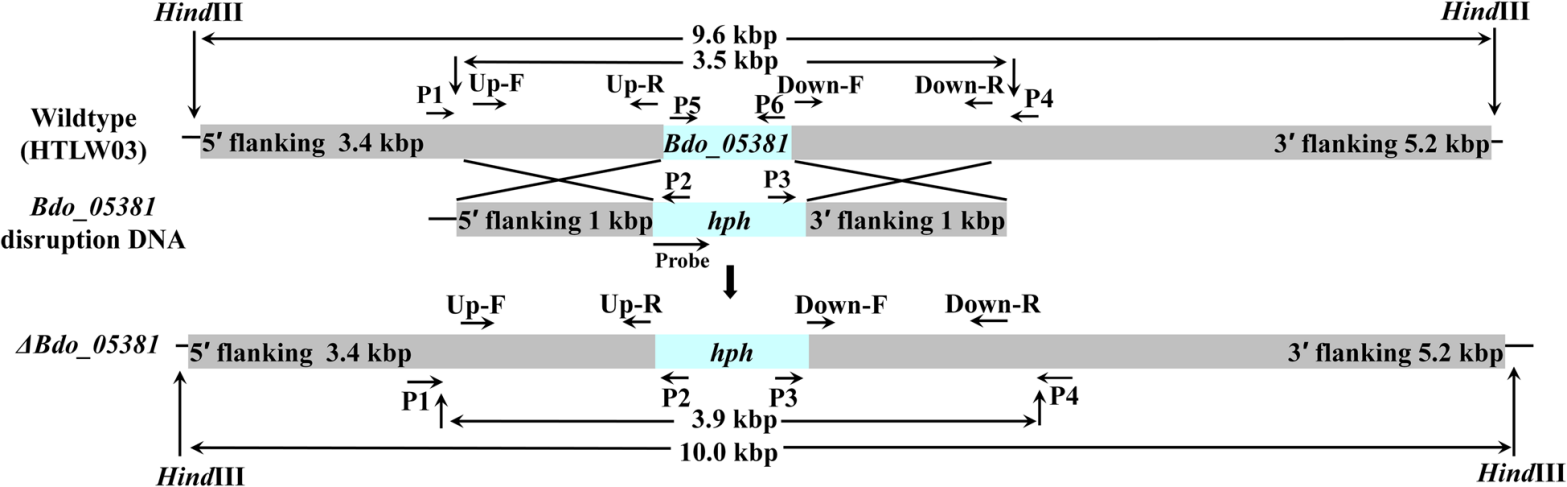
Homologous recombination strategy and primers used for verifying the *Bdo_05381-*disrupted transformants. Arrows indicate the positions of the primers used for constructing the plasmid and for verifying the transformants by PCR. The restriction enzyme digestion sites are marked*.* The *hph* gene controlled by the *trpC* promoter was used as the resistance marker. The 5′ and 3′ fragments flanking the target gene were linked to the sequences upstream and downstream of *hph*, respectively, to disrupt the target gene. A 500-bp *hph* gene probe was used for a Southern blot analysis

The hygromycin-resistance gene (*hph*) activated by *trp*C promoter was used for resistance selection. For homologous replacement, a 1000 bp fragment of 5′ flanking sequences of the target gene was fused to the 5′ terminus of *hph*, and a same size of 3′ flanking sequences of the target gene was fused to the 3′ teminus. When the homologous recombination happens on both upstream and downstream, the target gene is replaced by *hph* (Fig. 1). The correct GD transformant will have only one *hph* gene locus. When PCR verification is used to examine the GD transformants, about 1-kbp PCR products of upstream (with P1 and P2) and downstream (with P3 and P4) will be amplified. The open reading frame fragment (ORF) will be absent in the PCR product with P5 and P6. The PCR product of GD transformants will be 400-bp longer than wild type with P1 and P4 (Fig. 1).

**Fig. 2:**
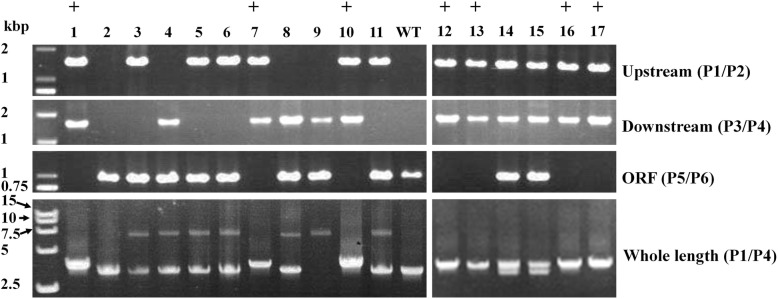
Verification of *Bdo_05381-*disrupted *B. dothidea* HTLW03 transformants by PCR. Transformants were analyzed with four PCR amplifications. The lanes marked with “+” correspond to the correct GD transformants with *Bdo_05381* upstream and downstream fragments that were the correct size, an amplicon that was 400 bp longer than that of *Bdo_05381*, and no open reading frame fragment

When 10 μg of GHR cassette was used for transformation, 39 transformants were obtained for *Bdo_05381* (Table 1). The transformation efficiency was 3.9 ± 0.71 per 10^6^ protoplasts. *B. dothidea* is a coenocytic fungus. When PCR verification was applied, various patterns of PCR results were observed in PCR verification of GD. If there are two or multiple haploid nuclei in one cell, and only random insertion happened, the results of PCR verification will be same as the wild type, like No. 2 in Fig. 2. If homologous recombination happens in all nucleus, and the target gene is fully disrupted, result of PCR will be same as the No. 1, 7, 10, 12, 13, 16 and 17 in Fig. 2. If homologous recombination happens in partial nucleus, result of PCR will be the superposition by wild type and successfully disrupted nucleus, result of PCR will be same as the No. 14 and 15 in Fig. 2. If recombination happens only on upstream or downstream fragment in partial nuclei (Additional file 1: Figure S1), the pattern of PCR products will be superposition by wild type and incomplete disrupted nuclei, like No. 3–6, 8 and 11 in Fig. 2. If homologous recombination happens only on downstream fragment in all nuclei, result of PCR will be the same as No. 9 in Fig. 2.

**Table 1:** Disruption efficiency of transforming *B. dothidea* HTLW03 with plasmid or cassette

Gene ID	DNA	No. of transformants	No. of correct transformants	Transformation efficiency (No. of transformants/10^6^ protoplasts)	GD efficiency (No. of transformants/10^7^ protoplasts)
*Bdo_05381*	Cassette	39	9	3.9 ± 0.71	9.0 ± 2.1 a
	Plasmid	22	1	2.2 ± 0.28	1.0 ± 1.4 b

Molar mass of 4.34 pmol transforming plasmid or disruption cassette were used. Values are means ± SD from two independent experiments. Values followed with different letters are significantly different according to ANOVA and Duncan’s method (*P* < 0.05)

Among the 39 transformants, nine showed upstream (amplified with P1 and P2) and downstream fragments (amplified with P3 and P4) of *Bdo_05381* of the correct size, 400-bp longer amplicons than *Bdo_05381* (amplified by P1 and P4), and the absence of the ORF fragment (amplified with P5 and P6) (Figs. 1, 2), like No. 1, 7, 10, 12, 13, 16 and 17 in Fig. 2, indicating that *Bdo_05381* was successfully disrupted*.* Thus, the GD efficiency was 9.0 ± 2.1 transformants per 10^7^ protoplasts. When the same molar-mass of GHR plasmid was used, which contained the same 5′ and 3′ flanking sequences and *hph,* the GD efficiency was decreased to 1.0 ± 1.4 transformants per 10^7^ protoplasts (Table 1). This result showed that using a GHR cassette gave higher GD efficiency than using a GHR plasmid*.*

### Optimization of GHR cassette quantity for higher GD efficiency

**Fig. 3:**
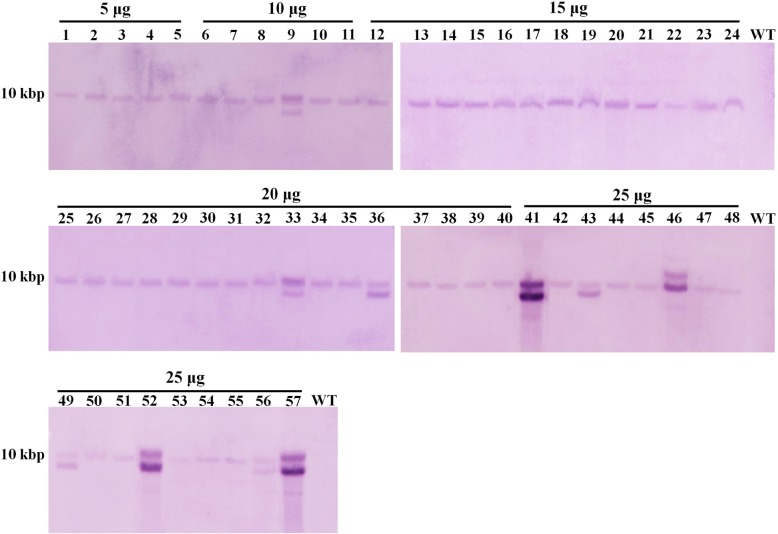
Southern blot of 57 *Bdo_05381-*disrupted *B. dothidea* HTLW03 transformants obtained with varying cassette DNA amounts. Lanes with only one band correspond to the transformants with one *hph* locus, whereas lanes with more than one band correspond to the transformants with redundant *hph* insertions

We next tested the effect of GHR cassette quantity on GD efficiency targeting *Bdo_05381*. When various amounts of GHR cassette DNA were used for transformation, 5–17 GD transformants were obtained (Table 2). The transformation efficiency ranged from 2.7 ± 0.42 to 5.7 ± 0.42 transformants per 10^6^ protoplasts, and the GD efficiency varied from 0.5 ± 0.14 to 1.7 ± 0.21 per 10^6^ protoplasts. There was no significant difference among the systems containing 15–25 μg of GHR cassette. When the obtained transformants were analyzed with southern blotting and a *hph* gene probe, two out of 16 and seven out of 17 transformants obtained with 20 and 25 μg of GHR cassette, respectively, showed more than one specific hybridization band, while all the transformants obtained with 15 μg of GHR cassette showed only one specific hybridization band (Fig. 3). This result indicated that when the amount of GHR cassette used in the transformation system was more than 15 μg, the frequency of random insertion increased. Thus, a transformation system with 15 μg of GHR cassette was used when the protocol was applied to disrupt a gene in another strain of *B. dothidea*, ZY7. In total, five correct *Bdo_05381* disruption transformants were obtained. All the transformants obtained had only one *hph* gene locus (Additional file 2: Figure S2). Moreover, we applied this protocol to disrupt another gene (*Bdo_02540*) in strain HTLW03 and successfully obtained one correct disruption transformant (Additional files 3, 4: Figures S3, S4), although the GD efficiency was lower than that of *Bdo_05381* (Table 2). Thus, this method showed great potential for GD in *B. dothidea*.

**Table 2:** Disruption efficiency of transforming *B. dothidea* HTLW03 strain with cassette DNA

Gene ID	Quantity of DNA (μg)	No. of transformants	No. of correct transformants	Transformation efficiency (No. of transformants/10^6^ protoplasts)	GD efficiency (No. of transformants/10^6^ protoplasts)
*Bdo_05381*	5	27	5	2.7 ± 0.42	0.5 ± 0.14 bc
	10	38	6	3.8 ± 0.56	0.6 ± 0.28 bc
	15	54	13	5.4 ± 0.85	1.3 ± 0.14 ab
	20	57	16	5.7 ± 0.49	1.6 ± 0.14 a
	25	54	17	5.4 ± 0.42	1.7 ± 0.21 a
*Bdo_02540*	15	22	1	2.2 ± 0.56	0.1 ± 0.14 c

Values are means ± SD from two independent experiments. Values followed with different letters are significantly different (P < 0.05) according to ANOVA and Duncan’s method

### Phenotype of GD tranformants

**Fig. 4:**
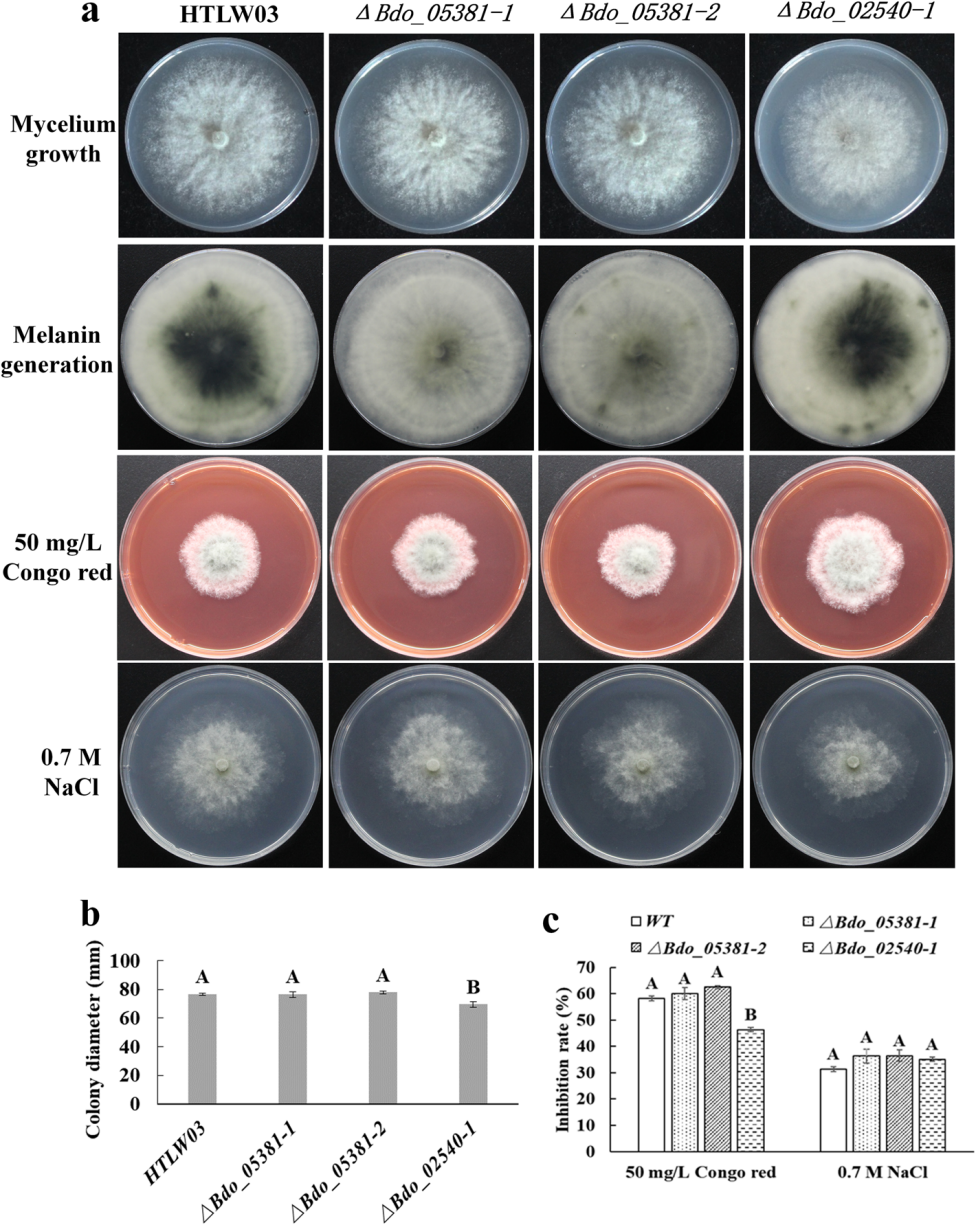
Phenotype of GD *B. dothidea* HTLW03 transformants. **a** Colony morphology of *ΔBdo_05381–1*, *ΔBdo_05381–2*, and *ΔBdo_02540–1* in Petri plates containing PDA medium or PDA medium supplemented with 50 mg/L Congo red and 0.7 M NaCl. Accumulation of melanin in 6-day-old cultures is presented*.***b** Comparison of the colony diameters of 60-h-old cultures on PDA medium. **c** Inhibitory effects of 50 mg/L Congo red and 0.7 M NaCl on the WT control and GD transformants. Bars represent the standard error of two independent experiments. The significance of the differences between the GD transformants (*ΔBdo_05381–1*, *ΔBdo_05381–2*, and *ΔBdo_02540–1*) and the WT control was analyzed based on an ANOVA and Duncan’s method. Different letters represent significant differences (*P* < 0.01)

The gene expression analysis confirmed that *Bdo_05381* and *Bdo_02540* were up-regulated during melanin accumulation stage (Additional file 5: Figure S5) [25], but their function has not been studied. We speculated that the *Bdo_05381* and *Bdo_02540* disruption transformants would be altered in melanin accumulation and tolerance to environmental stress. Therefore, colony morphology and tolerance of the disrupted transformants to Congo red and NaCl were investigated. The *ΔBdo_05381–1* and *ΔBdo_05381–2* showed similar growth rate and resistance to Congo red and NaCl as the WT, but produced less melanin. The *ΔBdo_02540–1* had a slower growth rate and a stronger resistance to Congo red, but showed similar melanin accumulation and resistance to NaCl as the WT (Fig. 4).

## Discussion

In this study, we established an efficient GD protocol for the woody plant pathogen *B. dothidea* by using a GHR cassette and optimizing the quantity of transformed DNA in the system. When using 15 μg of GHR cassette per 10^6^ protoplasts, we achieved high GD efficiency (1.3 ± 0.14 per 10^6^ protoplasts) with low random insertion. We successfully applied this protocol to disrupt genes in two *B. dothidea* strains.

The results of this study showed that when using the same molar mass of GHR cassette and plasmid DNA in the transformation system, the GHR cassette gave much higher GD efficiency than the plasmid. This result agrees with findings in *V. dahliae* [26], *Lecanicillium lecanii* [27], *Fusarium graminearum* [28], *Candida albicans* [29] and *Acremonium implicatum* [30]. Moreover, although the GD efficiency increased when the GHR cassette quantity was increased from 5 to 25 μg per 10^6^ protoplasts, multiple insertions occurred when the GHR cassette quantity was more than 15 μg per 10^6^ protoplasts. Besides, GD efficiency also varied with target genes. In strain HTLW03, GD efficiency of *Bdo_02540* is significantly lower than *Bdo_05381.*

GD by homologous recombination has great potential for studying gene functions, and has been successfully used in many filamentous fungi. In this study, we obtained GD transformants of *Bdo_05381* and *Bdo_02540* using homologous recombination successfully*. Bdo_05381*-disrupted transformants produced less melanin, whereas the *Bdo_02540*-disrupted transformant showed a slower growth rate and a stronger resistance to Congo red. The function of these two genes remains to be investigated.

The difficulties encountered in *B. dothidea* GD through homologous recombination are mainly due to (1) low transformation efficiency, which is significantly lower than in other filamentous fungi such as *V. dahliae* [20] and *M. oryzae* [31]; (2) and the coenocytic situation in its hyphae and conidia. To obtain more transformants, we increased the protoplast concentration (from the commonly used 10^6^ to 10^7^/mL). Increasing the amount of transformants increased the chance to get correct GD transformants.

In coenocytic fungi, it is only possible to obtain GD transformants containing a single integrated nucleus in one protoplast [32]. Therefore, increasing the percentage of monokaryotic protoplasts is very important to obtain pure GD transformants. In this study, we extended the lysis time for protoplasts to 3.5 h, which is favorable for monokaryotic protoplasts [23]. In fungi with monokaryotic conidia, transformants can be purified via single-spore isolation [33], but this method is not applicable for fungi like *B. dothidea*, which has coenocytic conidia. In this study, we applied three rounds of purification through single-hyphal-tip isolation successively on potato dextrose agar (PDA) plates containing 30 μg/mL hygromycin B, as purified transformants benefited from high selective pressure [33, 34].

## Conclusion

In conclusion, the established GD protocol makes genetic manipulation of the coenocytic woody plant pathogen *B. dothidea* possible. This method provides an efficient approach for researching the pathogenic mechanism of this pathogen and may be applicable to other coenocytic fungi as well.

## Methods

### Strain and culture conditions

Two virulent *Botryosphaeria dothidea* strains, HTLW03 and ZY7, were used in this study. The strain HTLW03 was isolated from Chinese flowering crabapple in Shandong Province, while ZY7 was isolated from apple in Henan Province. The strains were stored in 30% glycerin at 4 °C in the Mycology Lab, College of Plant Protection, China Agricultural University. The WT and transformant strains were maintained on PDA (potato 200 g/L, dextrose 20 g/L, agar 15 g/L) plates [23]. To test the abiotic stress sensitivities of each strain, culture blocks (5 mm in diameter) were placed on PDA plates containing Congo red (50 mg/L) or NaCl (0.7 M). Colony diameters were measured after incubating the cultures at 26 °C in darkness for 60 h. The melanin accumulation in cultures was observed after 6-day incubation. All experiments were performed twice, and each treatment was completed in triplicate.

### Protoplast isolation and purification

Protoplast isolation and purification were performed as described in Chen et al. [23] with some modifications. Briefly, six culture plugs (ca. 0.5 × 0.5 cm) were placed in 100 mL potato dextrose broth (potato 200 g/L, dextrose 20 g/L) and incubated at 26 °C in a shaker (50 rpm) in darkness for 42 h. The mycelial pellets were centrifuged at 5000 *g* for 10 min in 50 mL tubes, and then washed twice with 0.7 M NaCl solution. One gram wet pellet was added to 5 mL Lysing Enzyme solution (6% Lysing Enzyme, Sigma, St. Louis, MO, USA; 0.7 M NaCl) and the tubes were incubated at 32 °C for 3.5 h at 60 rpm for protoplast release. Then, the mixture was filtered through 3-layer lens paper to remove any hyphal fragments, and the infiltrate was centrifuged for 15 min at 4000 rpm. The supernate was discarded and protoplasts in the tube were washed once with STC (1 M sorbitol, 0.1 M Tris-HCl pH 8.0, 0.1 M CaCl_2_) solution to remove residual lysing enzyme and then re-suspended in STC solution. The protoplast concentration was measured with a hemocytometer and diluted to 1 × 10^7^/mL, and the tubes were stored on ice.

### Construction of the GD vector

The gene sequence including the upstream and downstream regions was retrieved from the Joint Genome Institute (JGI) fungal genome resource (https://genome.jgi.doe.gov/programs/fungi/index.jsf). We generated a GHR plasmid containing a hygromycin-resistance gene (*hph*) with flanking sequences of the target gene (Fig. 1). The 5′ and 3′-flanking sequences of *Bdo_05381* were amplified with Ex *Taq* (TaKaRa, Dalian, China) and Up-F/R and Down-F/R primers, respectively (Additional file 6: Table S1). The amplicons were purified using an Axygen gel extraction kit (Axygen, Union City, CA, USA). The *hph* gene with the *trpC* promoter was inserted into the t-clone site of the pMD19-T vector (TaKaRa, Dalian, China). Then, the 5′ and 3′-flanking sequences (1000 bp each) were inserted respectively into the *Sac*I and *Hind*III enzyme digestion sites using a Vazyme one-step cloning kit (Vazyme, Nanjing, China). The GHR plasmid was transferred into *Escherichia coli* using the heat-shock method [35].

### Preparation of DNA

Genomic DNA was extracted using CTAB solution (2% CTAB, 100 mM Tris-HCl pH 8.0, 1.4 M NaCl, 2% polyvinylpyrrolidone, 20 mM EDTA pH 8.0) following the DNA extraction protocol described by Kuhad et al. [36].

The *E. coli* was cultured in Luria-Bertani broth (tryptone 10 g/L, yeast extract 5 g/L, NaCl 10 g/L) with 100 μg/mL ampicillin at 37 °C with 180 rpm shaking for 16 h. Then, the plasmid DNA was extracted by the alkaline lysis method [37]. The GHR cassette (the *hph* gene with the 5′ and 3′ flanking sequences of *Bdo_05381*) was amplified using the primers Up-F and Down-R (Additional file 6: Table S1, Fig. 1) from the GHR plasmid. The PCR product was precipitated with 0.7 volumes isopropanol and 0.1 volumes 3 M NaAc (pH 5.2). The concentration and quality of the DNA was analyzed using a Nanodrop 2000 Spectrophotometer (Thermo Fisher Scientific, CA, USA).

### PEG-mediated transformation

The PEG-mediated transformation protocols described by Fitzgerald et al. [38] and Zhang et al. [27] were used with some modifications. Various amounts of GHR plasmid or GHR cassette and 100 μL protoplasts were placed in 1.5 mL sterile tubes, and then mixed adequately. After the mixture was kept on ice for 30 min, 1 mL PEG solution (40% PEG Sigma, 0.1 M CaCl_2_, 0.1 M Tris-HCl pH = 8.0) was carefully added dropwise. The tube was then rolled and mixed until the liquids were combined. After incubation at 30 °C for 30 min, the protoplasts in PEG solution were centrifuged for 8 min at 2000 *g*. The supernate was discarded and the pellets were re-suspended with 1 mL regeneration broth (RB) medium (0.52 g/L KCl, 0.52 g/L MgSO_4_·7H_2_O, 0.25 g/L KH_2_PO_4_, 6 g/L NaNO_3_, 1.2 M sorbitol, 1% dextrose, 100 μg/mL ampicillin) gently. The suspension was transferred to a 50 mL tube and diluted using 2 mL RB medium. The tube was maintained in a 26 °C incubator for 12 h.

To screen for resistant colonies, 20 mL regeneration agar medium (RB medium, 1% agar, 15 μg/mL hygromycin B) was added to the tube and it was shaken gently. The medium was then distributed into two 9-cm plates and incubated at 26 °C for 24 h. Subsequently, 15 mL PDA containing 25 μg/mL hygromycin B was placed on the surface of the plates and they were incubated at 26 °C in darkness. Colonies rising to the top layer of the medium were transferred to new PDA plates containing 30 μg/mL hygromycin B and sub-cultured for three generations to get pure homologous disruption transformants.

### Transformant verification

The primers designed for analysis are shown in Additional file 6: Table S1. The gene upstream sequence was amplified using the P1 and P2 primers and the downstream sequence was amplified with the P3 and P4 primers. Then, the P5 and P6 primers were used to amplify the open reading frame. The PCR was performed in a 25-μL volume containing 2.0 mM MgCl_2_, 1.0 U of r*Taq* polymerase (TaKaRa, Dalian, China), 200 μM each dNTP, 0.4 μM each specific primer, and 10–20 ng genomic DNA. The PCR parameters were 94 °C for 5 min, followed by 30 cycles (94 °C for 30 s, 58 °C for 30 s, and 72 °C for 1 min), and a final extension of 72 °C for 10 min in a Veriti™ 96-Well Thermal Cycler (Thermo Fisher Scientific, CA, USA). The *hph* gene was amplified with the P1 and P4 primers using the above PCR protocol, except the polymerase was LA *Taq* (TaKaRa, Dalian, China) and the extension time was 5 min. The transformation and disruption efficiencies were calculated using the equations:

Transformation efficiency = colonies on the hygromycin B plate/ protoplasts quantity.

Disruption efficiency = correct GD transformants by PCR analysis/ protoplasts quantity.

### Southern blot

Southern blot analysis was used to detect the number of insertion loci of the transformed DNA. The sequence of the resistance gene (ca. 500 bp) was used for probe labeling. The genomic DNA was digested with the *Hind*III enzyme (TaKaRa, Dalian, China). Then, the genomic DNA fragments were separated through 0.8% agarose gel and transferred onto a nylon membrane based on the capillary principle with 20 × SSC (3 M NaCl, 0.3 M Na-citrate, pH 7.0). The digested genomic DNA was probed using a 500-bp *hph* gene DNA fragment labeled following the instructions of the DIG High Prime DNA Labeling and Detection Starter Kit I (Roche, Mannheim, Germany).

### qRT-PCR analysis

The qRT-PCR was used to detect the expression pattern of *Bdo_05381* and *Bdo_02540* in mycelium. The mycelia cultured on PDA plates for 3, 6, and 9-day were collected. RNA was extracted using RNAiso Plus (TaKaRa, Dalian, China), and reversely transcribed with an oligo (dT)18 primer using Reverse Transcriptase M-MLV (TaKaRa, Dalian, China) following the manufacturer’s instruction. The *Actin* was used as internal control. The PCR was performed in qPCR Tower2.0 (Analytik Jena, Germany) using TB Green Premix DimerEraser™ qPCR mix (TaKaRa, Dalian, China) following method described by Tao et al. [39]. The results were analyzed using the 2^-ΔΔct^ method [40].

### Statistical procedures

Every treatment contained 10 transformation systems and each repeat contained five systems. Data were analyzed using the SPSS software and significant differences were analyzed according to one-way analysis of variance (ANOVA) and Duncan’s method.

## Supplementary information

**Additional file 1: Fig. S1.** Predicted PCR products when the homologous recombination occurred only with the upstream or downstream fragment. Solid lines represent the homologous recombination, whereas dashed lines indicate a lack of homologous recombination. **a** Homologous recombination involving the upstream fragment. **b** Homologous recombination involving the downstream fragment.

**Additional file 2: Fig. S2.** Verification of the *Bdo_05381* GD *B. dothidea* ZY7 transformants. Five *Bdo_05381*-disrupted transformants were analyzed with four PCR amplifications and a Southern blot. **a** All transformants comprised the correct upstream (left) and downstream (right) fragments. **b** Absence of the ORF fragment in the transformants. **c** Longer whole-length fragment in the transformants than in the WT control. **d** Southern blot analysis of the *hph* insertion loci. Lanes 1–5 in **a**–**d** correspond to five GD transformants. Lane 6 corresponds to the linearized plasmid (11 kbp).

**Additional file 3: Fig. S3.** Genomic details regarding *Bdo_02540.* Arrows represent the primers used for constructing the plasmid and for verifying the transformants by PCR. The restriction enzyme digestion sites are indicated.

**Additional file 4: Fig. S4.** Verification of *Bdo_02540* GD *B. dothidea* HTLW03 transformants. The *Bdo_02540* GD transformant (*ΔBdo_02540–1*) was analyzed with four PCR amplifications and a Southern blot. **a***ΔBdo_02540–1* had the correct upstream and downstream fragments, lacked the ORF fragment, and had a longer whole-length fragment than the WT control. **b** Southern blot analysis of the *hph* insertion loci.

**Additional file 5: Fig. S5.***Bdo_02540* and *Bdo_05381* expression in mycelium cultured for 3, 6, and 9 days on PDA. The expression in 3-day-old culture with mycelium appeared whitish on the underside of the plate was used as the control. The relative expression levels were calculated according to the 2^−ΔΔCt^ method. Bars represent the standard error of three replicates. Different letters represent significant differences according to an ANOVA and Duncan’s method (*P* < 0.05).

**Additional file 6: Table S1.** Primers used for vector construction and transformants analysis. The lowercase represented the homologous sequence flanking the restriction sites of *Sac*I and *Hind*III.

## Data Availability

The datasets used and analyzed during the current study are available from the corresponding author on reasonable request.

## Abbreviations

ANOVA: One-way analysis of variance
GD: Gene disruption
GHR: Gene homologous recombination
ORF: Opening reading frame
PDA: Dextrose agar medium
PEG: Polyethylene glycol
RB: Regeneration broth
