# Supplementary material for: An efficient gene disruption method for the woody plant pathogen Botryosphaeria dothidea
Source: BMC Biotechnol. 2020 Mar 5;20:14. doi: 10.1186/s12896-020-00608-z (PMC7059327; doi:10.1186/s12896-020-00608-z)
Supplement: Supplementary file 1 — Additional file 1: Fig. S1. Predicted PCR products when the homologous recombination occurred only with the upstream or downstream fragment. Solid lines represent the homologous recombination, whereas dashed lines indicate a lack of homologous recombination. a Homologous recombination involving the upstream fragment. b Homologous recombination involving the downstream fragment. [file 12896_2020_608_MOESM1_ESM.pdf]

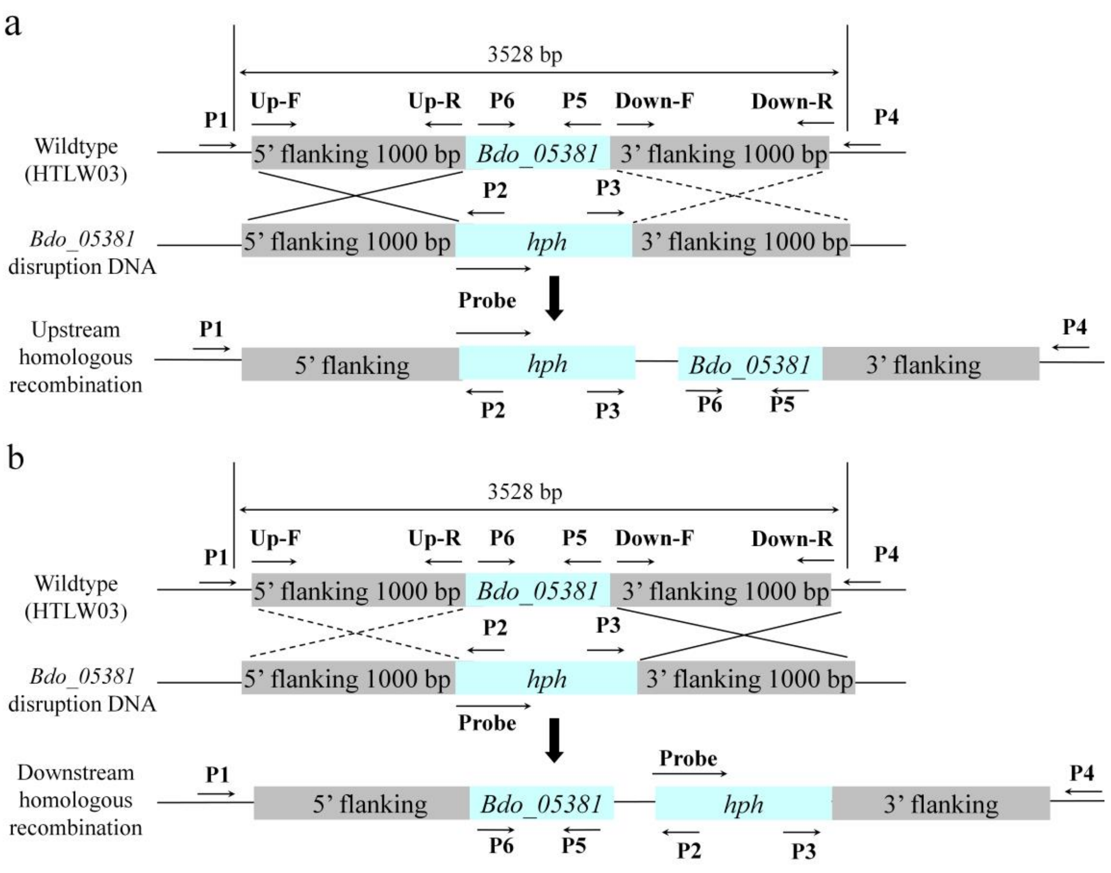

Fig. S1 Predicted PCR products when the homologous recombination occurred only with the upstream or downstream fragment

Solid lines represent the homologous recombination, whereas dashed lines indicate a lack of homologous recombination. **a** Homologous recombination involving the upstream fragment. **b** Homologous recombination involving the downstream fragment.
