# Supplementary material for: An efficient gene disruption method for the woody plant pathogen Botryosphaeria dothidea
Source: BMC Biotechnol. 2020 Mar 5;20:14. doi: 10.1186/s12896-020-00608-z (PMC7059327; doi:10.1186/s12896-020-00608-z)
Supplement: Supplementary file 2 — Additional file 2: Fig. S2. Verification of the Bdo_05381 GD B. dothidea ZY7 transformants. Five Bdo_05381-disrupted transformants were analyzed with four PCR amplifications and a Southern blot. a All transformants comprised the correct upstream (left) and downstream (right) fragments. b Absence of the ORF fragment in the transformants. c Longer whole-length fragment in the transformants than in the WT control. d Southern blot analysis of the hph insertion loci. Lanes 1–5 in a–d correspond to five GD transformants. Lane 6 corresponds to the linearized plasmid (11 kbp). [file 12896_2020_608_MOESM2_ESM.pdf]

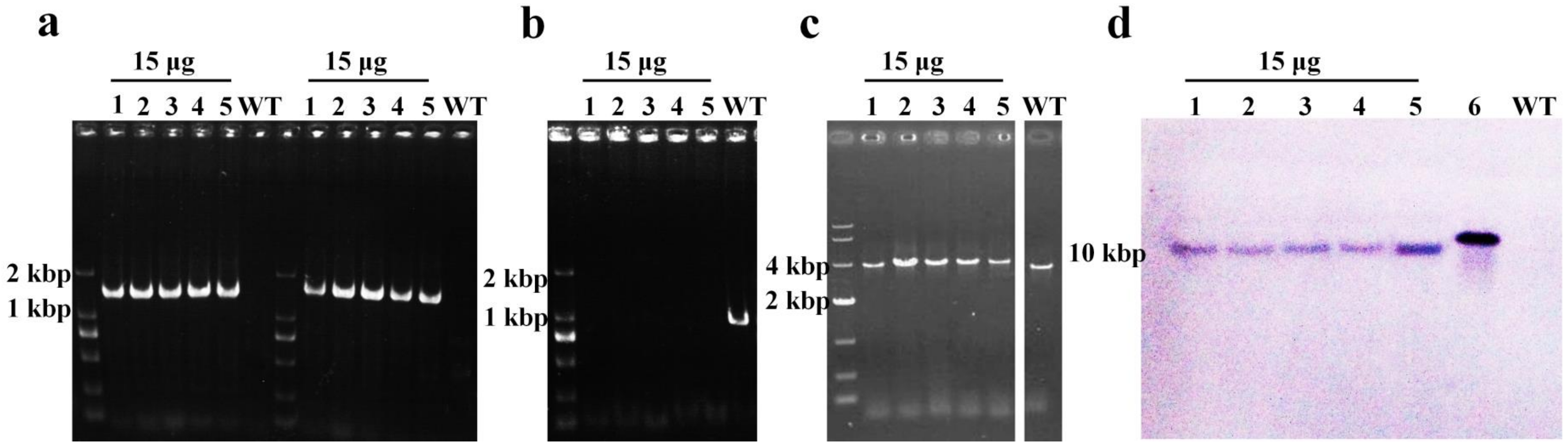

Fig. S2 Verification of the *Bdo_05381* GD *B. dothidea* ZY7 transformants

Five *Bdo_05381*-disrupted transformants were analyzed with four PCR amplifications and a Southern blot. **a** All transformants comprised the correct upstream (left) and downstream (right) fragments. **b** Absence of the ORF fragment in the transformants. **c** Longer whole-length fragment in the transformants than in the WT control. **d** Southern blot analysis of the *hph* insertion loci. Lanes 1–5 in **a**–**d** correspond to five GD transformants. Lane 6 corresponds to the linearized plasmid (11 kbp).
