# Supplementary material for: An efficient gene disruption method for the woody plant pathogen Botryosphaeria dothidea
Source: BMC Biotechnol. 2020 Mar 5;20:14. doi: 10.1186/s12896-020-00608-z (PMC7059327; doi:10.1186/s12896-020-00608-z)
Supplement: Supplementary file 3 — Additional file 3: Fig. S3. Genomic details regarding Bdo_02540. Arrows represent the primers used for constructing the plasmid and for verifying the transformants by PCR. The restriction enzyme digestion sites are indicated. [file 12896_2020_608_MOESM3_ESM.pdf]

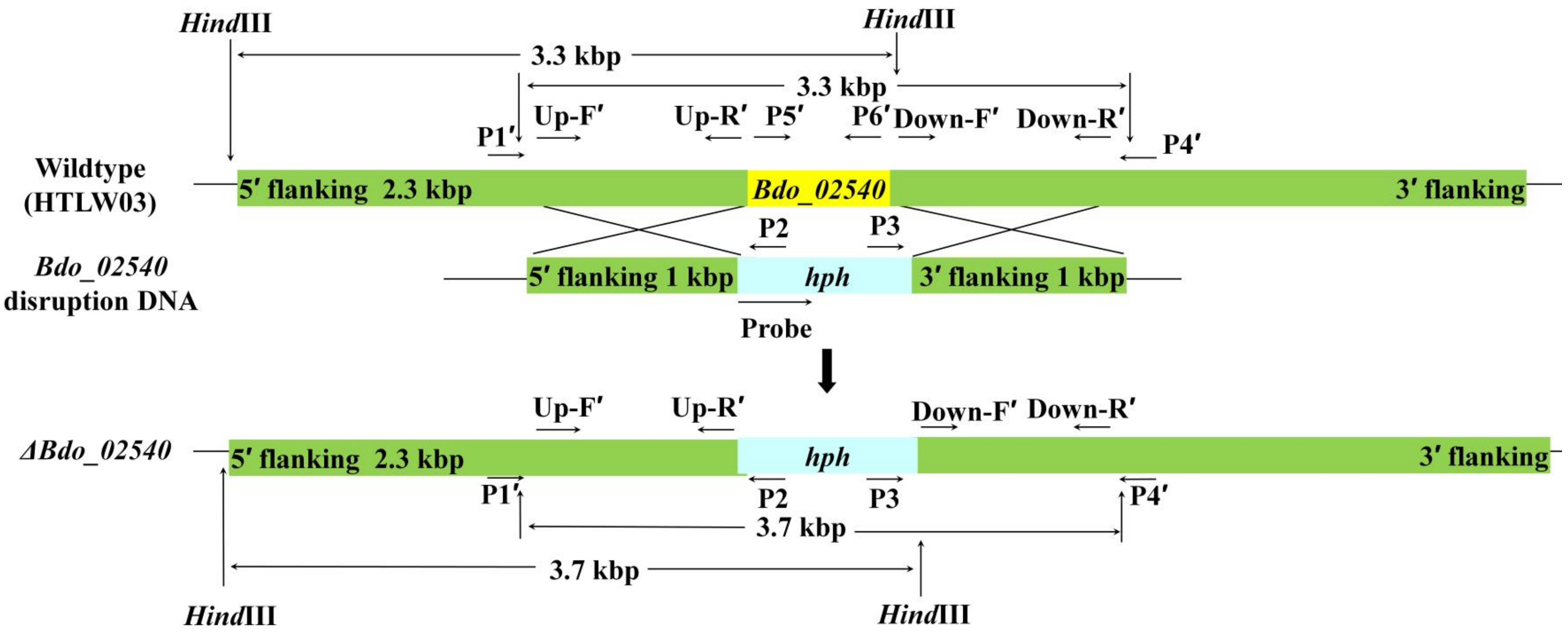

Fig. S3 Genomic details regarding *Bdo_02540*

Arrows represent the primers used for constructing the plasmid and for verifying the transformants by PCR. The restriction enzyme digestion sites are indicated.
