# Supplementary material for: An efficient gene disruption method for the woody plant pathogen Botryosphaeria dothidea
Source: BMC Biotechnol. 2020 Mar 5;20:14. doi: 10.1186/s12896-020-00608-z (PMC7059327; doi:10.1186/s12896-020-00608-z)
Supplement: Supplementary file 4 — Additional file 4: Fig. S4. Verification of Bdo_02540 GD B. dothidea HTLW03 transformants. The Bdo_02540 GD transformant (ΔBdo_02540–1) was analyzed with four PCR amplifications and a Southern blot. aΔBdo_02540–1 had the correct upstream and downstream fragments, lacked the ORF fragment, and had a longer whole-length fragment than the WT control. b Southern blot analysis of the hph insertion loci. [file 12896_2020_608_MOESM4_ESM.pdf]

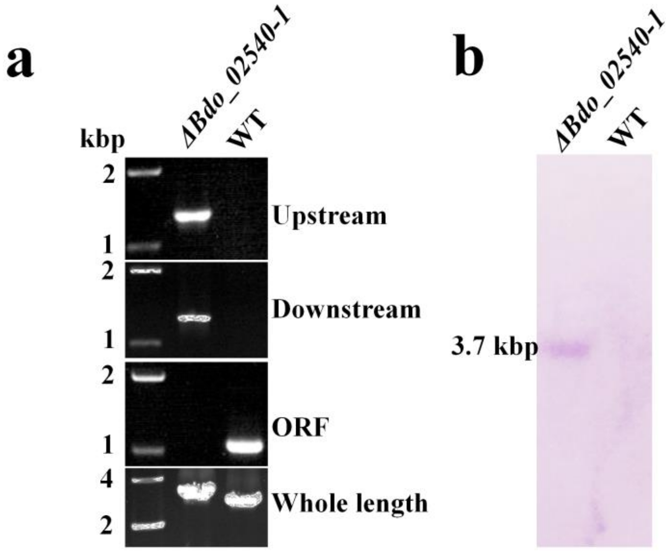

Fig. S4 Verification of *Bdo_02540* GD *B. dothidea* HTLW03 transformants

The *Bdo_02540* GD transformant (*ΔBdo_02540-1*) was analyzed with four PCR amplifications and a Southern blot. **a** *ΔBdo_02540-1* had the correct upstream and downstream fragments, lacked the ORF fragment, and had a longer whole-length fragment than the WT control. **b** Southern blot analysis of the *hph* insertion loci.
