# Supplementary material for: An efficient gene disruption method for the woody plant pathogen Botryosphaeria dothidea
Source: BMC Biotechnol. 2020 Mar 5;20:14. doi: 10.1186/s12896-020-00608-z (PMC7059327; doi:10.1186/s12896-020-00608-z)
Supplement: Supplementary file 5 — Additional file 5: Fig. S5.Bdo_02540 and Bdo_05381 expression in mycelium cultured for 3, 6, and 9 days on PDA. The expression in 3-day-old culture with mycelium appeared whitish on the underside of the plate was used as the control. The relative expression levels were calculated according to the 2−ΔΔCt method. Bars represent the standard error of three replicates. Different letters represent significant differences according to an ANOVA and Duncan’s method (P < 0.05). [file 12896_2020_608_MOESM5_ESM.pdf]

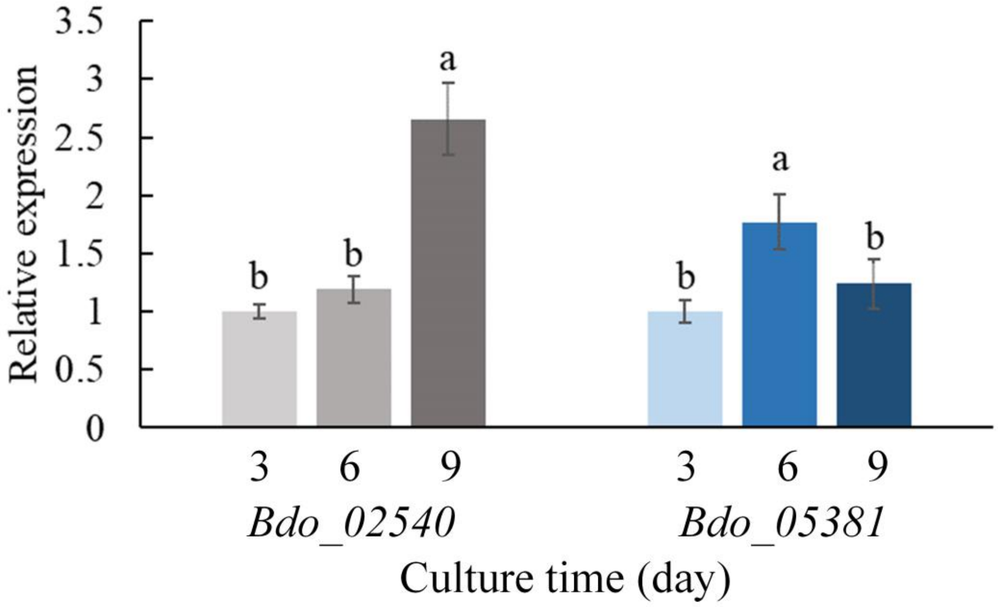

Fig. S5 *Bdo_02540* and *Bdo_05381* expression in mycelium cultured for 3, 6, and 9 days on PDA

The expression in 3-day-old culture with mycelium appeared whitish on the underside of the plate was used as the control. The relative expression levels were calculated according to the $2^{-\Delta\Delta Ct}$ method. Bars represent the standard error of three replicates. Different letters represent significant differences according to an ANOVA and Duncan's method ($P < 0.05$).
