# Supplementary material for: An efficient gene disruption method for the woody plant pathogen Botryosphaeria dothidea
Source: BMC Biotechnol. 2020 Mar 5;20:14. doi: 10.1186/s12896-020-00608-z (PMC7059327; doi:10.1186/s12896-020-00608-z)
Supplement: Supplementary file 6 — Additional file 6: Table S1. Primers used for vector construction and transformants analysis. The lowercase represented the homologous sequence flanking the restriction sites of SacI and HindIII. [file 12896_2020_608_MOESM6_ESM.doc]

**Table S1** Primers used for vector construction and transformants analysis

| Application | Primer name | Sequence (5’-3’) |
| --- | --- | --- |
| GHR plasmid construction of *Bdo_05381* | Up-F | acggccagtgaattcgagctcCAATGCCTGCAACACCATGCCGA |
|  | Up-R | ggatccccgggtaccgagctcAGAGCGACGTATTCGGTCGGTTG |
|  | Down-F | gacctgcaggcatgcaagcttCTGGATGATTAGCTGCCATGCTGG |
|  | Down-R | gaccatgattacgccaagcttCGAGCGTCATCAGTCCGTACTGAT |
| GHR plasmid construction of *Bdo_02540* | Up-F′ | acggccagtgaattcgagctcGTCATGTCGGGAACCAGCGAG |
|  | Up-R′ | ggatccccgggtaccgagctcGGCTGAGAAGCTGTAGTTTAGTTGG |
|  | Down-F′ | gacctgcaggcatgcaagcttCTTGCGGCAATGTCAGGTGAATG |
|  | Down-R′ | gaccatgattacgccaagcttAAGCTCGGTCCGCGACCTGAA |
| Verification of GD transformants | P1 | TGCTCGAGCGCATTGGAGGAG |
|  | P2 | GGTCGTTCACTTACCTTGCTTGAC |
|  | P3 | CTCTATCAGAGCTTGGTTGACGG |
|  | P4 | GCAAGTACCAGCAGCTCTCCAG |
|  | P5 | ATGAAGGTTGCTACTTTTCTTCTCGG |
|  | P6 | AGAAGCAACAAACAGGTCAGCAGC |
| Verification of GD transformants | P1′ | GTCGTACCAGTTGAGTTTCCATCTC |
|  | P4′ | TTGGACAGAACCGGCAGCAACTT |
|  | P5′ | ATGAAGGTCCAAGTTGCTGCTTTC |
|  | P6′ | TCACAACTTCGCAATGACCTCATCCTC |
| DIG probe labeling | Probe-F | CTCCCGATTCCGGAAGTGCTTGA |
|  | Probe-R | CCACGGCCTCCAGAGAAGATGTT |
| qRT-PCR analysis | Actin-F | CAACTGGGACGACATGGAGAAGATTTG |
|  | Actin-R | GATCTGGGTCATCTTCTCACGGTTG |
|  | 05381-F | CTTGACTTCTGCCACGACGAAGG |
|  | 05381-R | AGCTCCTCGAGGTCACGACGA |
|  | 02540-F | GACAACCTCTACACCAAGGATGGC |
|  | 02540-R | CATCAACTCCCTCTCAAGAGTCACG |

The lowercase represented the homologous sequence flanking the restriction sites of *Sac*I and *Hind*III.
